# Depression and incident alopecia areata and atopic dermatitis: A prospective cohort study

**DOI:** 10.1016/j.jdin.2026.05.028

**Published:** 2026-06-19

**Authors:** Dan Wang, Weisong Luo, Peizhi Deng, Yuzhou Huang, Jianyun Lu

**Affiliations:** a Department of Dermatology, The Third Xiangya Hospital, Central South University, Changsha, Hunan, China; b Postdoctoral Station of Medical Aspects of Specific Environments, The Third Xiangya Hospital, Central South University, Changsha, China

**Keywords:** alopecia areata, atopic dermatitis, depression, genetic susceptibility, inflammatory markers

*To the Editor:* Alopecia areata (AA) and atopic dermatitis (AD) are common chronic inflammatory skin diseases with substantial clinical and psychosocial burden.^1^^,^^2^ Depression is frequently observed in both conditions,^3^^,^^4^ yet prospective evidence integrating genetic susceptibility and inflammatory pathways remains limited. We examined whether baseline depression was associated with incident AA and AD in the UK Biobank and explored roles of genetic susceptibility and inflammatory markers.

Depression, AA, and AD were ascertained using linked health records. Cox proportional hazards models estimated adjusted hazard ratios (HRs). Polygenic risk scores (PRSs) for AA and AD were derived from published genome-wide association studies (Supplementary Tables I and II; Methods, available via Mendeley at https://data.mendeley.com/datasets/ck3mz5ym5f/1) and categorized into tertiles. Mediation analyses were performed for selected inflammatory factors. A negative-control outcome using kidney stones was conducted in the National Health and Nutrition Examination Survey.

During a median follow-up of 13.4 years, 180 incident AA and 2896 incident AD cases were identified (Table Ⅰ). Depression was associated with higher risks of incident AA (HR, 1.83; 95% CI, 1.22-2.74) and AD (HR, 1.53; 95% CI, 1.36-1.71) (Supplementary Table Ⅲ, available via Mendeley at https://data.mendeley.com/datasets/ck3mz5ym5f/1). Estimates for AA varied across subgroups and should be interpreted cautiously given the limited number of cases. No consistent subgroup differences were observed for AD (Supplementary Table Ⅳ, available via Mendeley at https://data.mendeley.com/datasets/ck3mz5ym5f/1).

**Table I:** Baseline characteristics of participants according to depression status

Baseline characteristics	Total participants (*n* = 416,983)	Depression		*P*
		No (*n* = 382535)	Yes (*n* = 34448)	
Age (y), mean ± SD	56.85 ± 8.00	56.95 ± 8.01	55.80 ± 7.77	<.001
Sex, *n* (%)				<.001
Female	225,362 (54.05)	202,788 (53.01)	22,574 (65.53)	
Male	191,621 (45.95)	179,747 (46.99)	11,874 (34.47)	
Average total household income before tax (€), *n* (%)				<.001
<18,000	80,044 (19.20)	70,050 (18.31)	9994 (29.01)	
18,000-30,999	92,521 (22.19)	84,629 (22.12)	7892 (22.91)	
31,000-51,999	94,895 (22.76)	87,906 (22.98)	6989 (20.29)	
52,000-100,000	73,436 (17.61)	69,174 (18.08)	4262 (12.37)	
>100,000	19,147 (4.59)	18,276 (4.78)	871 (2.53)	
Unknown	56,940 (13.66)	52,500 (13.72)	4440 (12.89)	
Education, *n* (%)				<.001
College or university degree	129,489 (31.05)	119,265 (31.18)	10,224 (29.68)	
Professional qualifications	116,654 (27.98)	107,122 (28.00)	9532 (27.67)	
A levels/AS levels or equivalent	22,282 (5.34)	20,358 (5.32)	1924 (5.59)	
O levels/GCSEs or equivalent	72,224 (17.32)	66,231 (17.31)	5993 (17.40)	
None of the above	72,810 (17.46)	66,306 (17.33)	6504 (18.88)	
Missing data	3524 (0.85)	3253 (0.85)	271 (0.79)	
Alcohol status				<.001
Current drinking	389,325 (93.37)	358,726 (93.78)	30,599 (88.83)	
Noncurrent drinking	27,301 (6.55)	23,521 (6.15)	3780 (10.97)	
Missing data	357 (0.08)	288 (0.07)	69 (0.20)	
Smoking				<.001
Current smoker	42,345 (10.16)	37,004 (9.67)	5341 (15.50)	
Noncurrent smoker	373,179 (89.50)	344,175 (89.97)	29,004 (84.20)	
Missing data	1459 (0.34)	1356 (0.36)	103 (0.30)	
Townsend index, mean ± SD	−1.54 ± 2.94	−1.59 ± 2.91	−0.98 ± 3.22	<.001
BMI (kg/m^2^), mean ± SD	27.41 ± 4.76	27.33 ± 4.68	28.25 ± 5.47	<.001
MET, min/wk	2665.30 ± 2659.99	2684.70 ± 2663.87	2442.31 ± 2604.73	<.001
Alopecia areata				<.001
Yes	180 (0.04)	151 (0.04)	29 (0.08)	
No	416,803 (99.96)	382,384 (99.96)	34,419 (99.92)	
Atopic dermatitis				<.001
Yes	2896 (0.69)	2548 (0.67)	348 (1.01)	
No	414,087 (99.31)	379,987 (99.33)	34,100 (98.99)	

*BMI*, Body mass index; *SD*, standard deviation; *MET*, Metabolic Equivalent of Task.

Higher genetic risk was associated with increased risks of AA and AD (Supplementary Table Ⅴ, available via Mendeley at https://data.mendeley.com/datasets/ck3mz5ym5f/1). Participants with both depression and high PRS had higher risks of AA (HR, 2.36; 95% CI, 1.18-4.73) and AD (HR, 2.24; 95% CI, 1.87-2.67), compared with those with no depression and low PRS. A gradient across PRS strata was observed, particularly for AD (Fig 1; Supplementary Table Ⅵ, available via Mendeley at https://data.mendeley.com/datasets/ck3mz5ym5f/1). Interactions between depression and PRS were significant (*P*-interaction = .001 for AA; <.001 for AD). Eosinophil percentage showed a statistically detectable but small mediating effect, which should be interpreted cautiously and does not support a biologic mechanism (Supplementary Tables Ⅶ and Ⅷ; Figs 2 and 3, available via Mendeley at https://data.mendeley.com/datasets/ck3mz5ym5f/1). Results were unchanged after excluding events within the first year (Supplementary Table Ⅸ, available via Mendeley at https://data.mendeley.com/datasets/ck3mz5ym5f/1). In the negative-control analysis, depression was inversely associated with kidney stones (OR, 0.66; 95% CI, 0.49-0.89) (Supplementary Table X, available via Mendeley at https://data.mendeley.com/datasets/ck3mz5ym5f/1).

**Fig 1:**
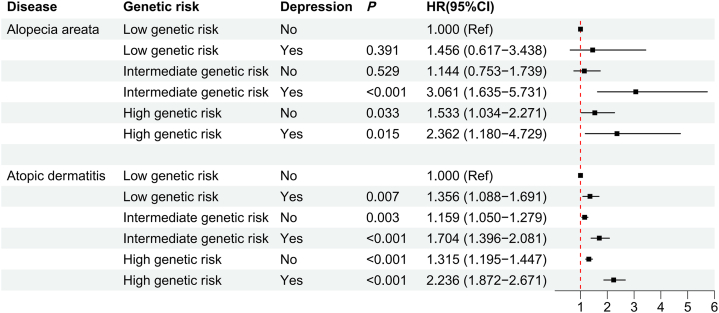
Joint associations of baseline depression and genetic susceptibility with the risk of incident alopecia areata (AA) and atopic dermatitis (AD).

Baseline depression was associated with increased risks of incident AA and AD. Joint associations with PRS were observed, although these findings should be considered exploratory and require further validation. The negative-control finding may not be fully explained by differences in health care utilization. However, this analysis does not fully exclude health care–seeking or ascertainment bias, given its use of National Health and Nutrition Examination Survey rather than UK Biobank, its cross-sectional design, and reliance on self-reported outcomes.

Limitations include the small number of AA cases, baseline-only assessment of depression, and potential residual confounding. PRSs were based on a limited number of variants, categorized into tertiles, and lacked external validation, which may limit risk discrimination. The cohort was restricted to individuals of European ancestry. Mediation effects were small.

In conclusion, depression was associated with increased risks of incident AA and AD. Findings related to genetic susceptibility and inflammatory markers are exploratory and require further validation.

### Declaration of generative AI and AI-assisted technologies in the writing process

During the preparation of this work the authors used DeepSeek-V3.2 in order to improve readability and language. After using this tool, the authors reviewed and edited the content as needed and take full responsibility for the content of the published article.

## Conflicts of interest

None disclosed.
